# Antiviral effect and mechanism of Phillyrin and its reformulated FS21 against influenza

**DOI:** 10.1111/irv.13112

**Published:** 2023-03-01

**Authors:** Yan Chen, Cunjin Wu, Huifen Li, Harrison Powell, Allison Chen, Guodong Zhu, Weihong Cong, Li Fu, Andrew Pekosz, Sean X. Leng

**Affiliations:** ^1^ Department of Geriatrics The First Affiliated Hospital of Chongqing Medical University Chongqing China; ^2^ Department of Geriatrics The Second Hospital of Tianjin Medical University Tianjin China; ^3^ W. Harry Feinstone Department of Molecular Microbiology and Immunology Johns Hopkins Bloomberg School of Public Health Baltimore Maryland USA; ^4^ Division of Geriatric Medicine and Gerontology, Department of Medicine Johns Hopkins University School of Medicine Baltimore Maryland USA; ^5^ Johns Hopkins Center on Aging and Immune Remodeling Baltimore Maryland USA; ^6^ Guangzhou Geriatric Hospital Guangzhou China; ^7^ National Clinical Research Center for Chinese Medicine, Xiyuan Hospital China Academy of Chinese Medical Sciences Beijing China; ^8^ Dalian Fusheng Natural Medicine Development Co. Ltd. Dalian China

**Keywords:** antiviral effect, FS21, influenza, influenza RNA polymerase inhibition, Phillyrin

## Abstract

**Background:**

Influenza virus causes significant morbidity and mortality with pandemic threat. *Oleaceae Fructus Forsythiae* is a medicinal herb. This study aimed to investigate antiviral effect of Phillyrin, a purified bioactive compound from this herb, and its reformulated preparation FS21 against influenza and its mechanism.

**Methods:**

Madin–Darby Canine Kidney (MDCK) cells were infected by one of six influenza viruses: five influenza A viruses (IAVs: three H1N1 and two H3N2) and one influenza B virus (IBV). Virus‐induced cytopathic effects were observed and recorded under microscope. Viral replication and mRNA transcription were evaluated by quantitative polymerase chain reaction (qPCR) and protein expression by Western blot. Infectious virus production was assessed using TCID50 assay, and IC50 was calculated accordingly. Pretreatment and time‐of‐addition experiments with Phillyrin or FS21 added 1 h before or in early (0–3 h), mid (3–6 h), or late (6–9 h) stages of viral infection were performed to assess their antiviral effects. Mechanistic studies included hemagglutination and neuraminidase inhibition, viral binding and entry, endosomal acidification, and plasmid‐based influenza RNA polymerase activity.

**Results:**

Phillyrin and FS21 had potent antiviral effects against all six IAV and IBV in a dose‐dependent manner. Mechanistic studies showed that both suppressed influenza viral RNA polymerase with no effect on virus‐mediated hemagglutination inhibition, viral binding or entry, endosomal acidification, or neuraminidase activity.

**Conclusions:**

Phillyrin and FS21 have broad and potent antiviral effects against influenza viruses with inhibition of viral RNA polymerase as the distinct antiviral mechanism.

## 1 INTRODUCTION

Influenza A and B viruses (IAV and IBV) circulate and cause seasonal epidemics worldwide,^1^ and IAV can cause pandemics in humans.^2^ Seasonal influenza imposes significant morbidity and mortality each year with especially high health burden to older adults.^3^ Hemagglutinin (HA) and neuraminidase (NA) are two major viral surface glycoproteins. HA is critical for viral binding to host cells as it binds to sialic acid receptors on the cell surface at the initiation of viral infection.^2^ For most influenza viruses, the acidic pH inside the endosomes after internalization of virions by endocytosis, or endosomal acidification, is also critical for viral entry to the host cells.^2^, ^4^ Influenza viral ribonucleoprotein (vRNP) complexes composed of viral RNA genome, nucleoprotein (NP), and viral RNA polymerases including subunits polymerase basic protein‐1 and ‐2 (PB1 and PB2), and polymerase acidic protein (PA), are all transported into the nucleus where viral replication and transcription occur. NA cleaves terminal sialic acid from host cell surface glycans, leading to the release of viral particles.^2^

Vaccination is the primary public health measure for the prevention of infectious diseases, including influenza and COVID‐19. However, the effectiveness of current influenza vaccines is suboptimal.^5^ Hence, effective antiviral agents for chemoprophylaxis and treatment of influenza are critically important for controlling this common and highly contagious respiratory viral infection. Three classes of antiviral agents have been approved worldwide for the treatment of influenza including ion channel blockers (the adamantanes: amantadine and rimantadine), NA inhibitors (oseltamivir, zanamivir, and peramivir), and an inhibitor of influenza cap‐dependent endonuclease (baloxavir).^6^ Because of widespread drug resistance, adamantanes have little clinical utility and are no longer recommended. Clinically important resistance to NA inhibitors and baloxavir has increasingly become a significant concern.^7^, ^8^ Favipiravir is a pro‐drug that inhibits viral RNA polymerase by targeting to its subunit PB1 after being converted by host enzymes and has been approved for use against emerging influenza viruses resistant to other antivirals in Japan.^9^ However, Goldhill and colleagues have reported that a specific mutation in the PB1 subunit confers resistance to this relatively new antiviral agent and, at the same time, a compensatory second mutation can restore influenza viral fitness.^10^ Taken together, the challenges of limited clinical effectiveness of influenza vaccines and increasing resistance to currently available antiviral agents highlight the urgent need for the development of novel antiviral therapies.

*Oleaceae Fructus Forsythiae* is widely used as an antipyretic herbal medicine in China, Japan, and Korea.^11^ This medicinal herb has been used as a Traditional Chinese Medicine (TCM) remedy for fever, pyrexia, and other flu‐like symptoms in China for centuries and is documented in Chinese Pharmacopeia as an active herbal component of 114 TCM medicinal preparations.^12^ Prior studies of *Oleaceae Fructus Forsythiae* employing its crude extract showed anti‐IAV H1N1 strain and its induced inflammatory cytokine responses in various degrees.^13^, ^14^ Phillyrin, also known as forsythin, is a major bioactive compound purified from *Oleaceae Fructus Forsythiae* with reported anti‐inflammatory and antioxidant properties.^15^, ^16^ One study in mice reported protective effects of Phillyrin against infection of mouse‐adapted IAV.^17^ FS21, a reformulated preparation that has been approved for the treatment of fever, pyrexia, and other influenza‐like symptoms in China since 2015, is composed of 90% purified Phillyrin with the remaining 10% of less purified components of *Oleaceae Fructus Forsythiae*. In this study, we comprehensively investigated the antiviral effect of Phillyrin and FS21 against six influenza viruses. We observed broad and potent antiviral effect of Phillyrin and FS21. Their antiviral effect is mediated by suppressing influenza viral RNA polymerase activity.

## 2 METHODS

### 2.1 Cells, viruses, plasmids, antibodies, and antiviral reagents

Madin–Darby Canine Kidney (MDCK, provided by Andrew Pekosz, a coauthor) and 293T cells (ATCC, MD, USA) were cultured in Dulbecco's modified Eagle's medium (DMEM; Thermo Fisher, CA). A549 (ATCC) were cultured in Earle's Minimum Essential Medium (EMEM; Thermo Fisher). IAV H3N2 (A/Victoria/361/2011, A/Udorn/1972), IAV H1N1 (A/California/07/2009, A/New Caledonia/20/1999 and A/WSN/1933), and IBV (B/Yamagata/2010) strains were provided by Andrew Pekosz. Viral stock was prepared as previously described^18^ in viral infectious media (DMEM supplemented with 0.3% bovine serum albumin and 1‐μg/ml trypsin, Sigma‐Aldrich, MO). Viral titer was measured by median tissue culture infectious dose (TCID_50_) assay as described below.

Plasmids (pcDNA3.1) expressing PB1, PB2, PA, and nuclear protein (NP) were obtained from Pekosz laboratory, so as RNA polymerase I expression vector pHH21 carrying an influenza virus‐like RNA encoding firefly luciferase (pHH21‐Luc). Antibodies were anti‐NP (GTX125989), anti‐PA (GTX125932), anti‐PB2 (GTX125925), and anti‐M1 (GTX125928), all from GeneTex, CO; anti‐β‐actin (58169, CST, MA); and horseradish peroxidase (HRP) conjugated anti‐rabbit and anti‐mouse secondary antibodies (7074 and 7076, CST).

Phillyrin and FS21, both provided by Dr. Li Fu (Dalian Fusheng Natural Medicine Development Co. Ltd., Dalian, Liaoning, China), were dissolved in DMEM with brief sonication as a stock solution (400 μg/ml) and stored at 4°C. Working solutions at desired concentrations were freshly prepared at the time of experiments. Oseltamivir and ribavirin were purchased from Sigma‐Aldrich.

### 2.2 Influenza infection

MDCK or A549 cells were pretreated with Phillyrin or FS21 at various concentrations for 1 h and inoculated with influenza virus at multiplicity of infection (MOI) 0.01. Virus inoculum was removed after 1‐h adsorption and replaced with viral infectious media with Phillyrin or FS21 at the same concentration. Culture supernatants or cell lysates were collected at indicated time points as hours post infection (hpi) for subsequent experiments.

### 2.3 Median tissue culture infectious dose (TCID_50_)

TCID_50_ was determined according to Reed and Muench.^19^ Briefly, MDCK cells were seeded onto 96‐well plate overnight, washed with phosphate buffered saline (PBS) twice, and then added with 10‐fold serial dilutions of viral supernatant in each of 8 duplicate wells. After culture for 3–5 days, cells were fixed with 4% paraformaldehyde (Sigma‐Aldrich) and stained with Napthol Blue‐Black (Sigma‐Aldrich) for 1 h. Inhibition rate was calculated as (TCID_50_virus − TCID_50_[virus + Phillyrin or FS21])/TCID_50_virus.

### 2.4 Time‐of‐addition experiments

After influenza virus infection, studies have shown that synthesis and export of viral genomes from the nucleus level out at 3hpi and release of virus particles occurs at 8hpi.^20^ As such, influenza virus life cycle can be divided into early (0–3 h), mid (3–6 h), and late (6–9 h) stages. To expand our kinetics analyses, time‐of‐addition experiments with Phillyrin or FS21 (100 μg/ml) were conducted in these three stages. Cellular viral replication (M vRNA) and M1 and M2 mRNA transcription were evaluated in the presence or absence of Phillyrin or FS21 at 0–9, 0–3, 3–6, and 6–9 h after virus inoculation. For example, to evaluate 3‐ to 6‐h time period, cells were infected with IAV (A/Victoria/361/2011) in viral inoculum containing no Phillyrin or FS21 at 0 h. Viral inoculum was removed, and cells were cultured in fresh medium for 3 h, and at 3hpi, Phillyrin or FS21 was added. They remained in the culture for 3 h and were removed at 6hpi. Cells were cultured in the fresh medium without virus or antivirals for an additional 3 h, and cell lysates were collected at 9hpi for quantitative polymerase chain reaction (qPCR) and Western blot analyses.

### 2.5 qPCR, Western blot, cytotoxicity, hemagglutination inhibition (HI), NA inhibition (NI), flow cytometry, and endosomal acidification

Methods for these experiments are described in detail in the Supporting Information.

### 2.6 Luciferase assay

A plasmid‐based reverse genetics system was used to evaluate influenza viral RNA polymerase activity using Bright‐GloTM Luciferase Assay System kit (E2610, Promega) as previously described.^20^, ^21^ With Lipofectamine™ 3000 Transfection Reagent (Thermo Fisher), 293T cells were transfected with plasmids expressing IAV (A/Udorn/1972) PB1, PB2, PA, NP, and pHH21‐Luc for 6 h and were subsequently treated with Phillyrin, FS21, or ribavirin (as positive control) for 24 h. Cell lysates were then harvested, and luciferase activity was measured using Wallac Victor (Perkin Elmer) according to manufacturer's instructions.

### 2.7 Statistical analysis

Experiment data were analyzed with SPSS 17.0 software. Results were presented as means ± standard deviations (SDs). Comparisons between means of two groups were made using an unpaired, two‐tailed Student's *t* test. Comparisons between means more than two groups were analyzed using one‐way analysis of variance (ANOVA). IC50 was calculated by nonlinear regression. Statistical significance was determined at *p* < .05 (*), *p* < .01 (**), or *p* < .001 (***).

## 3 RESULTS

### 3.1 Phillyrin and FS21 reduced influenza virus‐induced cytopathic effect (CPE)

**FIGURE 1:**
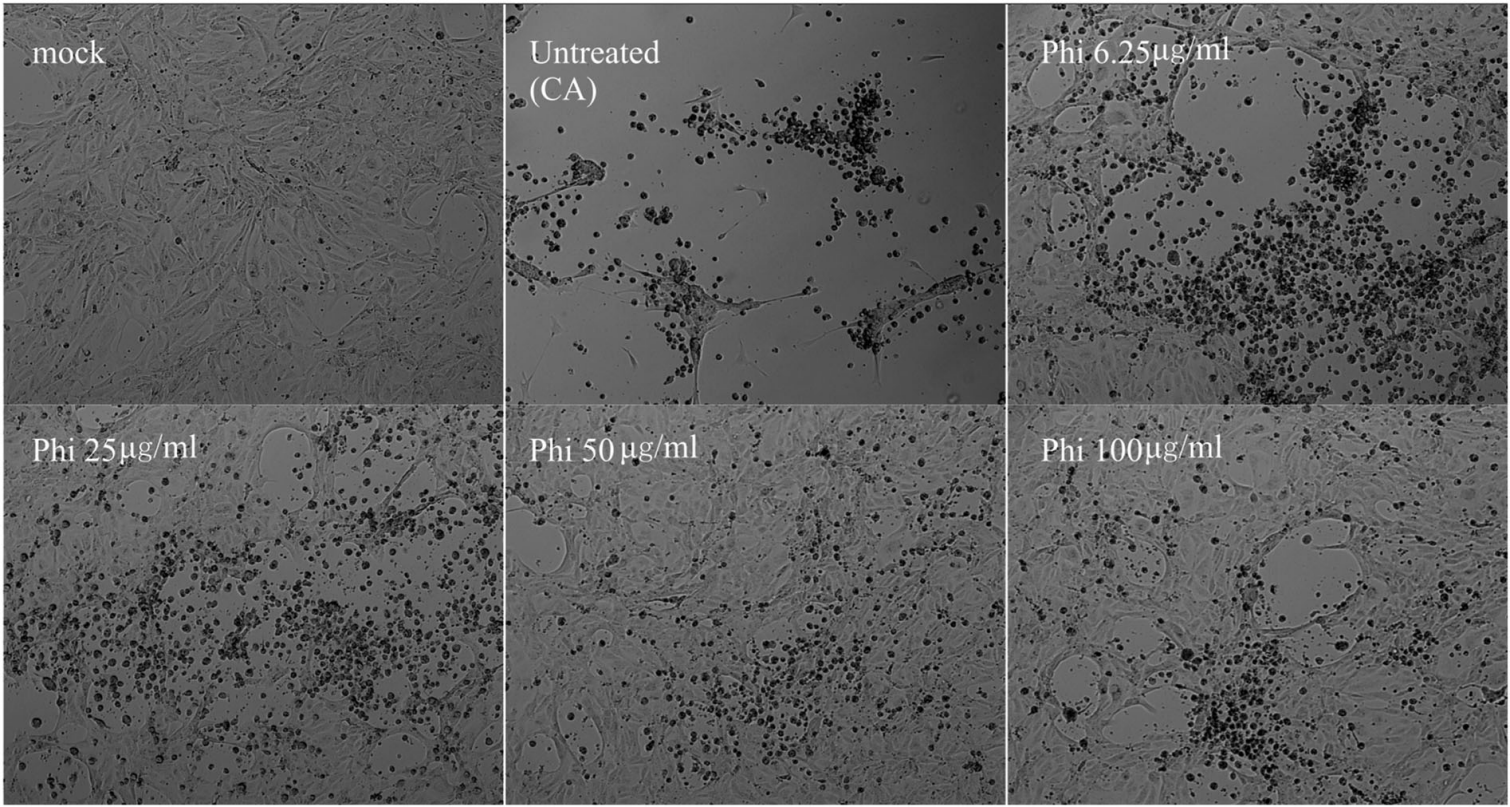
Phillyrin (“Phi”) reduced influenza virus‐induced cytopathic effect (CPE) by influenza A virus (IAV A)/California/07/2009. Madin–Darby Canine Kidney (MDCK) cells were pretreated with Phillyrin at indicated concentration for 1 h and inoculated with IAV A/California/07/2009 at multiplicity of infection (MOI) 0.01. After 1‐h adsorption, virus inoculum was removed and replaced with freshly prepared infectious media with Phillyrin at the same concentration. Virus‐induced CPE was recorded under microscopy at 48 h post infection (hpi). Results shown are from a typical experiment. “Mock” is the negative control with no influenza virus, cultured for the same time period in the presence of Phillyrin at 100 μg/ml.

MDCK cells were pretreated with Phillyrin or FS21 and infected with one of the six influenza viruses as described above. Direct microscopic observation at 48hpi showed that treatment with Phillyrin (Figure 1) or FS21 resulted in significant reduction of virus‐induced CPE by IAV H1N1 (A/California/07/2009) compared with untreated mock control. Similar results were observed with the other five influenza viruses when treated with Phillyrin or FS21 (data not shown).

Results from MTS assay demonstrate no cytotoxic effect of Phillyrin or FS21 at the concentrations tested for 48 or 72 h (Figure S1).

### 3.2 Phillyrin and FS21 suppressed influenza viral replication and protein expression as well as infectious virus production

**FIGURE 2:**
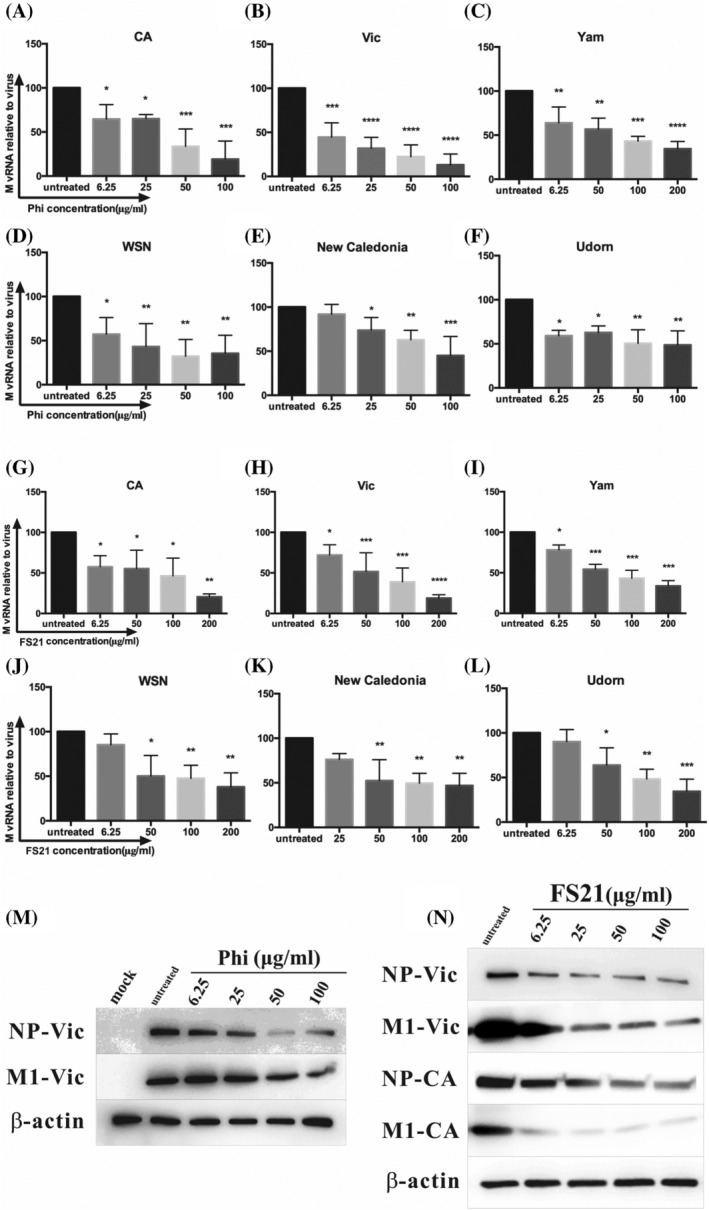
Phillyrin (“Phi”) and FS21 suppressed influenza viral replication and protein expression. Madin–Darby Canine Kidney (MDCK) cells were pretreated with Phillyrin or FS21 at indicated concentration for 1 h and inoculated with an influenza virus at multiplicity of infection (MOI) 0.01. After 1‐h adsorption, virus inoculum was removed and replaced with freshly prepared infectious media with Phillyrin or FS21 at the same concentration. Viral RNA (M vRNA) replication (A–L): Culture supernatants were collected at 48hpi, and RNA was isolated, and copies of M vRNA were determined using quantitative polymerase chain reaction (qPCR). % of M vRNA copies in virus‐infected samples in the presence of Phillyrin or FS21 relative to virus infection alone was calculated. The graphs represent the mean and standard deviation of three independent experiments of treatment by Phillyrin (A–F) or FS21 (G–L). Each graph represents one of the six influenza viruses tested as indicated (“CA”: A/California/07/2009; “Vic”: A/Victoria/361/2011; “Yam”: B/Yamagata/2010; “WSN”: A/WSN/1933; “New Caledonia”: A/New Caledonia/20/1999; and “Udorn”: A/Udorn/1972). Significant differences were identified by one‐way analysis of variance (ANOVA), *p < .05, **p < .01, ***p < .001, ****p < .0001; viral protein expression (M,N): Cell lysates were collected at 48hpi. Expression of influenza viral NP and M1 proteins was determined by Western blot. Results from typical experiments of Phillyrin treatment of “Vic” shown in (M) and FS21 treatment of “Vic” and “CA” shown in (N). β‐actin is shown as a loading control.

To determine the effect of Phillyrin and FS21 on influenza viral replication, copies of viral RNA genome (M vRNA) were assessed by qPCR in culture supernatant collected at 48hpi. The results showed potent suppressive effect of Phillyrin (Figure 2A–F) and FS21 (Figure 2G–L) on M vRNA replication of all six viruses in a dose‐dependent manner.

To examine the effect of Phillyrin and FS21 on viral protein expression, cell lysates collected at 48hpi were tested for influenza NP and M1 protein expression using Western blot. As demonstrated by results from a typical experiment (Figure 2M,N), Phillyrin or FS21 drastically reduced viral protein expression.

**FIGURE 3:**
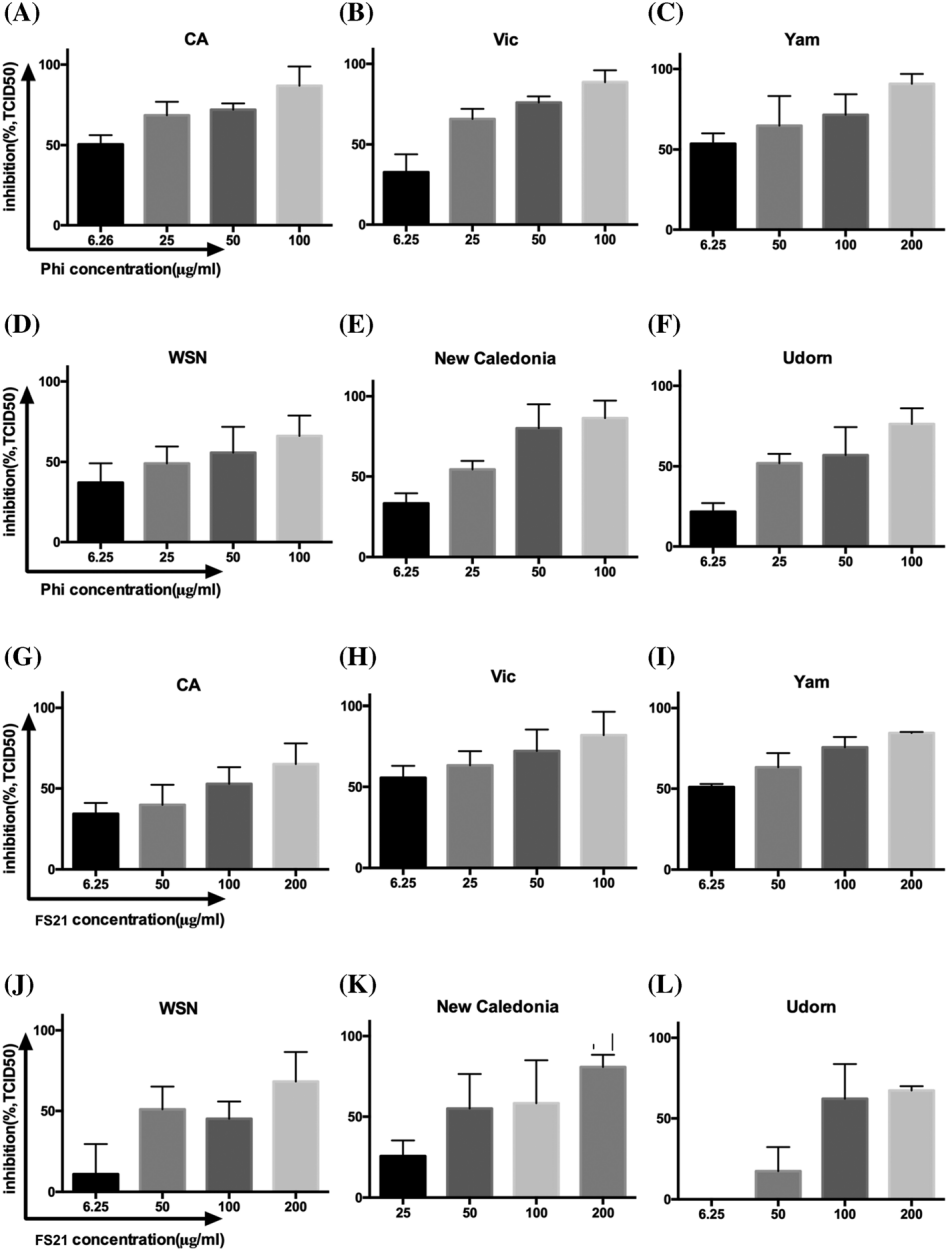
Phillyrin (“Phi”) and FS21 suppressed infectious virus production. Madin–Darby Canine Kidney (MDCK) cells were pretreated with Phillyrin or FS21 and infected with an influenza virus the same way as described in Figure 2. Culture supernatants were collected at 48hpi, and viral titers were determined by TCID_50_ assay. The graphs represent the mean and standard deviation of % inhibition TCID_50_ by the treatment of Phillyrin (A–F) or FS21 (G–L) from three independent experiments. Each graph represents one of the six influenza viruses as indicated.

To investigate effect of Phillyrin and FS21 on the production of infectious influenza viruses, viral titers in culture supernatants collected at 48hpi in the absence or presence of Phillyrin or FS21 at different concentrations were assessed using TCID_50_ assay. The results showed that Phillyrin (Figure 3A–F) and FS21 (Figure 3G–L) suppressed infectious virus production of all six influenza viruses tested in a dose‐dependent manner. Table 1 summarizes half maximal inhibitory concentrations (IC50s) of Phillyrin and FS21.

**TABLE 1:** Fifty percent inhibition concentration (IC50) against influenza viruses based on TCID_50_ assay^a^.

	IC50(μg/ml) against influenza virus					
	CA	Vic	Yam	WSN	New Caledonia	Udorn
Phillyrin	6.41 ± 1.35	13.20 ± 1.13	5.74 ± 1.86	24.52 ± 1.41	15.20 ± 1.19	27.59 ± 1.17
FS21	65.83 ± 1.48	7.58 ± 1.62	6.88 ± 1.41	78.49 ± 1.35	54.54 ± 1.32	99.30 ± 1.15

^a^ IC50 was calculated by nonlinear regression (curve fit) analysis.

Similar suppressive effects of Phillyrin or FS21 on influenza viral replication and infectious virus production were achieved in A549 cells as illustrated by the results from IAV A/Victoria/361/2011 infection shown in Figure S2.

Taken together, these results demonstrate that Phillyrin and FS21 have broad and potent suppressive effects on influenza virus‐induced CPE, viral RNA replication, viral protein expression, and infectious virus production.

### 3.3 Phillyrin and FS21 had no effect on virus HA‐mediated HI, virus binding and entry to, endosomal acidification, or viral release from host cells

Potential effect of Phillyrin and FS21 on HA‐mediated HI was evaluated. The results showed that Phillyrin or FS21 did not interfere with HA‐mediated HI by influenza virus as illustrated by the results from IAV A/Victoria/361/2011 infection (Figure S3a,b).

Flow cytometry analysis using fluorochrome‐conjugated anti‐influenza NP monoclonal antibody was employed to assess potential effect of Phillyrin and FS21 on viral binding and entry to the host cells. Because influenza virus can attach to the surface of the host cells (binding) at 4°C and viral entry occurs at 37°C, MDCK cells in the presence or absence of Phillyrin or FS21 (100 μg/ml) were incubated with one of the six viruses at MOI 5 for 1 h at 4°C (viral binding) and for additional 1.5 h at 37°C (viral entry). As an example illustrated by the results from a typical experiment (Figure S3c,d), Phillyrin had no effect on virus binding or entry into MDCK cells.

Next, potential effect of Phillyrin or FS21 on endosomal acidification was investigated using acridine orange to stain MDCK cells after virus infection in the presence or absence of Phillyrin or FS21. The results showed that infected cells maintained acidic staining despite Phillyrin or FS21 treatment, whereas bafilomycin A1 (positive control) abolished such acidic staining (Figure S3e).

NA activity was also evaluated using NI assay. Phillyrin or FS21 had no inhibitory effect on NA activity of influenza virus whereas oseltamivir (positive control) showed robust NI (Figure S3f–h).

Taken together, these results indicate that neither Phillyrin nor FS21 suppresses influenza virus binding to, entry of, or release from the host cells. In addition, Phillyrin or FS21 has no effect on endosomal acidification.

### 3.4 Kinetics analyses of suppressive effect of Phillyrin and FS21 on influenza viral replication, mRNA transcription, and protein expression

**FIGURE 4:**
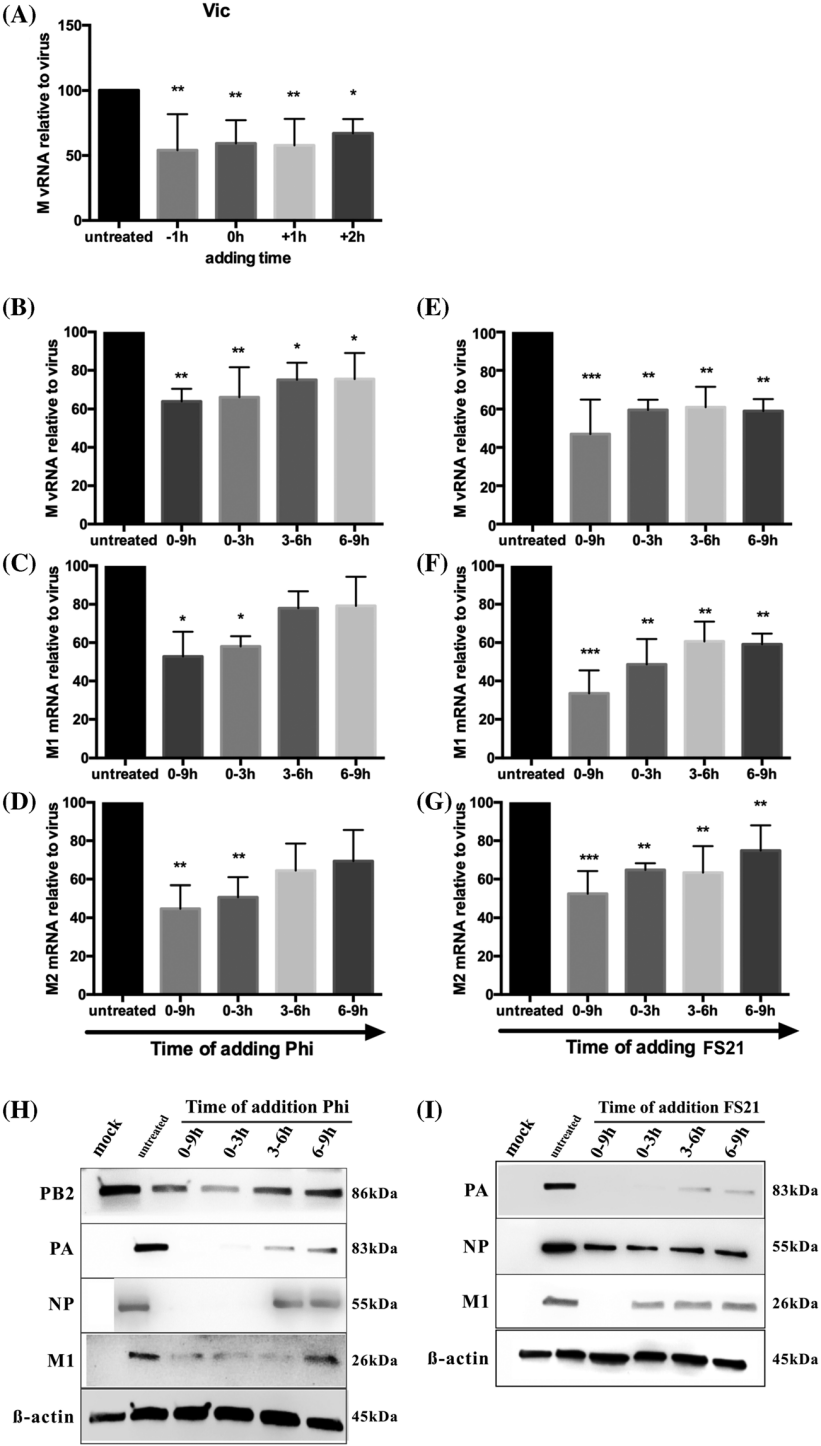
Kinetics analyses of suppressive effect of Phillyrin (“Phi”) and FS21 on influenza viral replication, mRNA transcription, and protein expression. Initial analysis of Phillyrin suppression of influenza viral replication: Madin–Darby Canine Kidney (MDCK) cells were infected with A/Victoria/361/2011 (“Vic”) at multiplicity of infection (MOI) 0.01 and treated with Phillyrin 1 h before viral inoculation (−1 h or pretreatment), at the same time (0 h), 1 or 2 h post‐infection (+1 or +2 h). Culture supernatants were collected at 48hpi and influenza viral RNA replication (“M vRNA”) was determined by quantitative polymerase chain reaction (qPCR) (A). Graph (A) represents the mean and standard deviation of the data from four independent experiments. Time‐of‐addition analyses of suppression of Phillyrin and FS21 of influenza viral replication, mRNA transcription, and protein expression (B–I): MDCK cells were inoculated with influenza A virus (IAV A)/Victoria/361/2011 at MOI 1.0 for 1 h at 37°C. Phillyrin at 100 μg/ml or FS21 at 100 μg/ml was added at each time intervals of 0–9, 0–3, 3–6, and 6–9 h post infection. Cell lysates were collected at 9hpi, and qPCR analyses were performed to evaluate M vRNA replication (B and E) and mRNA transcription of viral M1 and M2 genes (“M1 mRNA” and “M2 mRNA”, C, D, F, and G). The graphs represent the mean and standard deviation of the data from three independent experiments. *p < .05, **p < .01, ***p < .001. Graphs H–I illustrate suppressive effects of Phillyrin (H) and FS21 (I) on viral protein expression of influenza PA, NP, and M1 genes assessed by Western blot. β‐actin is shown as a loading control.

To examine the kinetics of antiviral effects of Phillyrin and FS21 against influenza, MDCK cells were infected with influenza virus at MOI 0.01 (1‐h adsorption in viral inoculum with no Phillyrin or FS21) and treated with Phillyrin or FS21 1 h before virus inoculation (−1 h or pretreatment), at the same time (0 h), or 1 or 2 h post‐infection (+1 or +2 h). Culture supernatants were collected at 48hpi, and influenza M gene vRNA replication was determined by qPCR. As illustrated by a representative set of results from a typical experiment of Phillyrin on IAV A/Victoria/361/2011 virus replication (Figure 4A), similar suppressive effects of Phillyrin and FS21 on virus replication were achieved at all these time points.

Influenza virus life cycle can be divided into early (0–3 h), mid (3–6 h), and late (6–9 h) stages. To expand our kinetics analyses, time‐of‐addition experiments with Phillyrin or FS21 (100 μg/ml) were conducted at these stages to evaluate the effects of Phillyrin and FS21 on vRNA replication (M region), mRNA transcription of M1 and M2 viral genes, and expression of viral proteins PA, NP, and M. As illustrated by a representative set of results from a typical experiment with infection of IAV A/Victoria/361/2011 (Figure 4B–G), Phillyrin and FS21 significantly inhibited influenza vRNA replication (Figure 4B,E, respectively) and mRNA transcription of viral M1 and M2 genes (Figure 4C,D and F,G, respectively) when it was present in any of the three stages. As expected, such suppressive effects were the strongest when Phillyrin or FS21 was present in the entire 0‐ to 9‐h period. They became weaker when Phillyrin or FS21 was added in a later stage (i.e., 6‐ to 9‐ or 3‐ to 6‐h period) but remained significant, except for the suppression of M1 and M2 mRNA transcription by Phillyrin that was no longer statistically significant (Figure 4C,D). Consistent with these results, Phillyrin and FS21 potently suppressed the expression of viral protein PA, NP, and M1, and such suppression reached almost completion when it was present in the entire 0‐ to 9‐h or early 0‐ to 3‐h periods (Figure 4H,I, respectively).

These results indicate that Phillyrin and FS21 suppress influenza virus replication as well as viral gene transcription and protein expression efficiently when included throughout the time of infection and with early treatment leading to greater effects than later treatment.

### 3.5 Phillyrin and FS21 suppressed viral RNA polymerase activity

**FIGURE 5:**
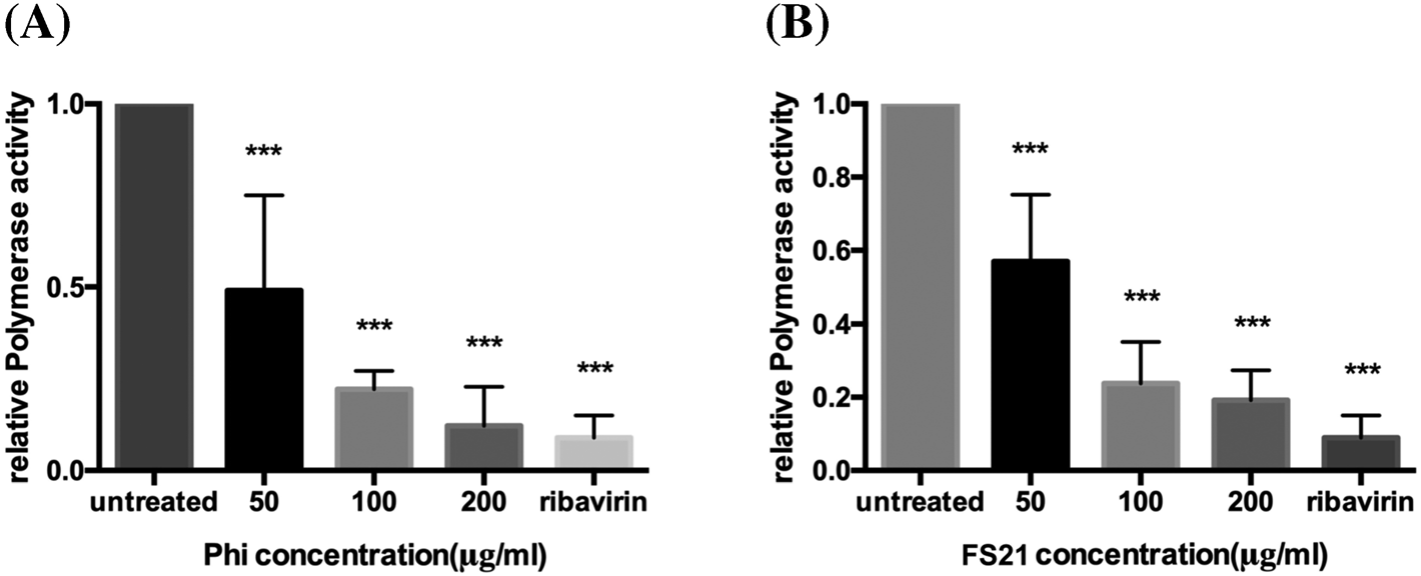
Phillyrin (“Phi”) and FS21 inhibit influenza RNA polymerase activity. 293T cells were transfected with plasmids expressing influenza A virus (IAV A)/Udorn/1972 polymerase proteins (PB1, PB2, and PA) and NP along with luciferase reporter plasmid (pHH21‐Luc) for 6 h. Transfected cells were cultured in the presence or absence of Phillyrin (A) or FS21 (B) at indicated concentration or ribavirin at 4.88 μg/ml (as a positive control) for an additional 24 h. Cell lysates were then harvested for luciferase activity measurement. The graphs represent the mean and standard deviation of data from four independent experiments. *p < .05, **p < .01, ***p < .001.

The effects of Phillirin and FS21 on viral RNA replication suggested that the treatments might target the influenza viral RNA polymerase. A plasmid‐based influenza RNA polymerase activity assay using a reporter gene^20^, ^21^ was employed to test this hypothesis directly. In these experiments, 293T cells were transfected with plasmids expressing IAV A/Udorn/1972 polymerase proteins (PB1, PB2, and PA) and NP along with a luciferase reporter plasmid (vNP‐Luc) for 6 h as described previously.^20^ Transfected cells were cultured in the presence or absence of Phillyrin or FS21 at various concentrations, or ribavirin at 4.88 μg/ml (as a positive control), for an additional 24 h. Cell lysates were then harvested for luciferase activity measurement. As shown in Figure 5, Phillyrin and FS21 reduced luciferase activity by 92% and 81.02%, respectively, at a similar level of reduction achieved by ribavirin, a known influenza RNA polymerase inhibitor.^22^ These results demonstrate that Phillyrin and FS21 can inhibit influenza viral RNA polymerase activity.

## 4 DISCUSSION

This study, for the first time, reports that Phillyrin, a major bioactive compound from a medicinal herb *Oleaceae Fructus Forsythiae* with 98% purity, and its reformulated preparation FS21 has potent antiviral effects against influenza infection in cell culture. Their antiviral effects are demonstrated through reduction of influenza virus‐induced CPE as well as suppression of viral genome RNA replication, mRNA transcription and protein expression, and infectious virus production. Such antiviral effects are broad as they are shown to be potent against all six influenza viruses tested. These viruses are genetically and antigenically distinct from each other and were isolated 40–70 years apart. Mechanistic studies indicate that neither Phillyrin nor FS21 interferes with HA‐mediated HI, virus binding or entry into the host cells, endosomal acidification, or NA activity. Instead, both suppressed influenza RNA polymerase activity. Our kinetics analyses demonstrate that these antiviral effects can be achieved by pretreatment or addition of Phillyrin or FS21 after virus inoculation up to 9 h, suggesting the potential utility of Phillyrin and FS21 for both chemoprophylaxis and treatment of influenza.

As expected, Phillyrin and FS21 have overall shown similar antiviral effects against influenza in this study. However, in our time‐of‐addition experiments, we observed that FS21 had more potent suppressive effects on viral replication, viral gene mRNA transcription, and protein expression than Phillyrin when it was added in mid or late stages of virus life cycle (Figure 4B–I). For example, inhibition of viral gene M1 and M2 mRNA transcription by Phillyrin became insignificant when it was added in 3–6 or 6–9 h after virus inoculation (Figure 4C,D) whereas such inhibition by FS21 remained statistically significant (Figure 4F,G). These results suggest the possibility of antiviral activity from less purified components with other bioactive compounds of *Oleaceae Fructus Forsythiae* in FS21 preparation.

The strengths of this study include (1) antiviral effects of Phillyrin and FS21 were thoroughly evaluated using multiple approaches, including influenza virus‐induced CPE, viral replication, viral gene mRNA transcription, and protein expression, as well as infectious virus production; (2) a total of six influenza viruses across IAV H1N1 and H3N2 as well as IBV were tested for their broad antiviral effects; (3) detailed kinetics analyses were conducted indicating potent antiviral effects during the entire virus life cycle including pretreatment. This observation has led us to investigating viral RNA polymerase activity as the underlying mechanism; and (4) after excluding the possibility of with interfering HA inhibition, viral entry, or NI, suppression of viral RNA polymerase activity was identified as the underlying molecular mechanism for the observed antiviral effects of Phillyrin and FS21. The anti‐viral RNA‐dependent RNA polymerase mechanism is particularly important and intriguing as it has therapeutic implications against other respiratory RNA viruses including SARS‐CoV‐2. For example, favipiravir has recently been approved by the US Food and Drug Administration (FDA) for the treatment of COVID‐19 under emergency authorization (UEA) although its clinical efficacy requires further evaluation.^23^

This study has limitations. For example, the plasmid‐based reverse genetics system employed in our study does not allow us to further identify the target site(s) of action of Phillyrin and FS21 in influenza viral RNA polymerase complex or its subunits. Influenza viral RNA polymerase complex requires PB1, PB2, and PA subunits, all of which contribute to the formation of the polymerase active site that is highly conserved in influenza virus evolution.^24^ Further studies are needed to determine whether Phillyrin and FS21 targets to this highly conserved viral RNA polymerase active site, which could greatly diminish the risk of drug resistance. Another limitation is that while Phillyrin is a major bioactive compound from *Oleaceae Fructus Forsythiae* with high purity, other bioactive compounds with antiviral properties against influenza in this medicinal herb might have been missed. For example, studies have shown antiviral effects of Forsythoside A and Labdane diterpenoids against IAV H1N1 strain.^25^, ^26^ As pointed out earlier, data presented in Figure 4C,D suggest such a possibility. Despite these limitations, findings from this study provide important pre‐clinical evidence strongly supportive of further development of Phillyrin and FS21 as promising novel agents for the chemoprophylaxis and treatment of influenza infection with a distinct antiviral mechanism. FS21 has shown excellent safety profile after its approval for clinical use to treat influenza‐like illness in China since 2015,^12^ highlighting the potential of successful development. Moreover, Phillyrin is reported to have anti‐inflammatory property,^15^ which may provide additional therapeutic benefits as local and systemic inflammation is considered one of the primary drivers of severe influenza disease and exacerbation of chronic conditions in other physiologic organ systems, particularly cardiovascular diseases.^27^ If their antiviral efficacy can be confirmed by studies in animal models and ultimately by clinical trials in humans, Phillyrin and FS21 will be great additions to our armamentarium for the chemoprophylaxis and treatment of influenza and possibly other respiratory RNA virus infections in clinical medicine. They may also be instrumental and can serve as therapeutic and research means addressing pathologies triggered by influenza and other respiratory RNA virus infections, such as systemic inflammation and related exacerbation of chronic diseases.

## AUTHOR CONTRIBUTIONS

**Yan Chen:** Data curation; investigation; methodology. **Cunjin Wu:** Investigation; methodology. **Huifen Li:** Investigation; methodology. **Harrison Powell:** Investigation; methodology. **Allison Chen:** Investigation; methodology. **Guodong Zhu:** Investigation; methodology. **Weihong Cong:** Writing ‐ original draft; writing ‐ review and editing. **Li Fu:** Conceptualization; resources. **Andrew Pekosz:** Resources; supervision; writing ‐ original draft; writing ‐ review and editing.

## CONFLICT OF INTEREST

The authors have no competing interests to declare.

### PEER REVIEW

The peer review history for this article is available at https://publons.com/publon/10.1111/irv.13112.

## Supporting information


**Figure S1.** No excessive cytotoxicity from Phillyrin (“Phi”) or FS21 treatment. MDCK cells were plated onto 96‐well plates at 1×10^4^ cells/well and treated with Phillyrin (a‐b) or FS21 (c‐d) at indicated concentration for 48 h or 72 h. MTS (3‐(4,5‐dimethylthiazol‐2‐yl)‐5‐(3‐carboxymethoxyphenyl)‐2‐(4‐sulfophenyl)‐2H‐tetrazolium) was added, and cells were incubated for additional 2 h. Absorbance at 490 nm was measured by BioTek Epoch microplate reader (Bio‐Tek, VT) to determine cell viability. The graphs represent the mean and standard deviation of data from 3 independent experiments. No statistical significance was observed between the treated and untreated groups.

**Figure S2.** Phillyrin (“Phi”) and FS21 suppressed influenza viral replication and infectious virus production in A549 cells. A549 cells were pretreated with Phillyrin or FS21 and infected with IAV A/Victoria/361/2011 virus the same way as described for MDCK cells in Figure 2. Culture supernatants were collected at 48hpi. Copies of M vRNA were determined using qPCR. % of M vRNA copies in virus‐infected samples in the presence of Phillyrin or FS21 relative to virus infection alone was calculated (a and b). Viral titers were determined by TCID50 assay (c and d). The graphs represent the mean and standard deviation from 3 independent experiments. *p < .05, **p < .01, ***p < .001, ****p < .0001.

**Figure S3.** Phillyrin (“Phi”) or FS21 had no effect on HA‐mediated hemagglutination inhibition (HI), influenza binding, entry, endosomal acidification, or neuraminidase activity. HI assays were performed using “Vic” and standard chicken RBC (cRBC) with or without pretreatment of Phillyrin (a) or FS21 (b) for 1 h. cRBCs in wells with no virus added or with hemagglutination inhibition sedimented and formed red buttons, whereas wells with positive hemagglutination had an opaque appearance with no sedimentation. Viral binding and entry (c‐d): MDCK cells were pretreated with Phillyrin (100 μg/ml) for 1 h and infected with “Vic” at MOI 5.0 at 4 °C for 1 h (c, binding), or for an additional 1.5 h at 37 °C, and virus was then removed from the cell surface by trypsin (d, entry). Virus particles were stained with anti‐NP monoclonal antibody at cell surface (c, binding) and intracellularly (d, entry) for Flow cytometry analysis. Endosomal acidification (e): MDCK cells infected with “Vic” were treated with or without Phillyrin or FS21 (“Vic + Phil” and “Vic + FS21”), or treated with 100 nM bafilomycin A1 (“Baf.A1”, positive control), and stained with 4 μg/ml acridine orange as described in Methods. NA inhibition (f‐h): NA activities from viruses “CA” treated with Phillyrin (f) or FS221 (h) or “Vic” treated with Phillyrin (g) at indicated concentrations are shown. Oseltamivir (“Oselt”) at 6.25 μg/ml was used as a positive control. The graphs represent the mean and standard deviation of data from 3 independent experiments. *p < .05, **p < .01, ***p < .001.

**Data S1.** Supporting Information

## Data Availability

The data that support the findings of this study are available from the corresponding author upon reasonable request.
