# Supplementary material for: Antiviral effect and mechanism of Phillyrin and its reformulated FS21 against influenza
Source: Influenza Other Respir Viruses. 2023 Mar 1;17(3):e13112. doi: 10.1111/irv.13112 (PMC9975791; doi:10.1111/irv.13112)
Supplement: Supplementary file 4 — Data S1. Supporting Information [file IRV-17-e13112-s003.docx]

# Methods for Quantitative PCR (qPCR), Western blot, cytotoxicity, hemagglutination inhibition (HI), neuraminidase assay (NA) inhibition, flow cytometry, and endosomal acidification

*Quantitative PCR (qPCR)*

Total RNA was extracted from culture supernatant or cell lysate routinely using QIAamp and Rneasy mini kits, respectively (Qiagen, CA), and reverse transcribed using SuperScript III reverse transcriptase (Thermo Fisher). For detection of IAV viral RNA genome in M gene region (M vRNA), a universal primer (5’-AGCAAAAGCAGG-3’) was used as reverse transcriptase (RT) primer. For detection of IBV M vRNA and message RNA (mRNA, M1 and M2 mRNA), random RT primer was used. cDNA was then quantified by qPCR using Universal SYBR Green Super mix kit (Bio-Rad, CA) and measured by CFX96C1000 Touch Real-Time PCR System Detector (Bio-Rad). The primers were as the following:

IAV M1 mRNA forward primer: 5’-TTCTAACCGAGGTCGAAAC-3’

IAV M1 mRNA reverse primer: 5’-AAGCGTCTACGCTGCAGTCC-3’

IAV M2 mRNA forward primer: 5’-GACCRATCCTGTCACCTCTGAC-3’

IAV M2 mRNA reverse primer: 5’-GGGCATTYTGGACAAAKCGTCTACG-3’

IAV M vRNA forward primer: 5’-TTCTAACCGAGGTCGAAAC-3’

IAV M vRNA reverse primer: 5’-AAGCGTCTACGCTGCAGTCC-3’

IBV M vRNA forward primer: 5’-CTAGGAACGCTCTGTGCTTTAT-3’

IBV M vRNA reverse primer: 5’-TAGCTGAGACCATCTGCATTTC-3’

*Western blot*

﻿Cell lysates were prepared and viral protein electrophoresis and quantification were performed as previously described^1^. Briefly, cell lysates were prepared with cell lysis buffer containing protease/phosphatase inhibitor cocktail (CST). Protein concentrations were determined by Bradford dye-binding (Bio-Rad). Protein extracts containing sodium dodecyl sulfate (SDS, Bio-Rad) and dithiothreitol (Bio-Rad) was boiled for 10 min at 95°C. Equal amount of protein extracts were loaded onto 4-12% Mini-Protean TGX gel (Bio-Rad) and transferred onto a polyvinylidene difluoride membrane (PVDF, Millipore, MA) at 120V for 90min. The membranes were then blocked in 5% nonfat milk for 1h at room temperature. After blocking, the blots were incubated with specific primary antibodies overnight and secondary antibodies at room temperature for 1h. Blots were enhanced with chemiluminescence (ECL, Kindle BioSciences, USA) and imaged by kwik Quant Imager (Kindle BioSciences).

*Cytotoxicity*

Cytotoxicity was tested by CellTiter 96® Aqueous One Solution cell proliferation assay (Promega, WI) ﻿as previously described^1^. Briefly, MDCK cells were plated in 96-well plates and treated with Phillyrin and FS21 at different concentrations. After culture for 48h and 72h, 20μL MTS (3-(4,5-dimethylthiazol-2-yl)-5-(3-carboxymethoxyphenyl)-2-(4-sulfophenyl)-2H-tetrazolium) was added to each well for 2h incubation. Absorbance at 490 nm was measured by BioTek Epoch microplate reader (Bio-Tek, VT).

*Hemagglutination inhibition (HI)*

Standardized chicken red blood cell (cRBC) solutions were prepared using 0.5% chicken red blood cells (CBT farms, Chestertown, MD). Viral supernatant in a series of 2-fold dilutions and chicken red blood cell (cRBC) were mixed together in round bottomed 96-well plate at an equal volume of 25μl for 1h at room temperature. cRBCs in wells with no viral supernatant added or negative hemagglutination sedimented and formed red buttons, whereas wells with positive hemagglutination had an opaque appearance with no sedimentation. Hemagglutination titers were calculated as hemagglutination units (HAU)/ml. HI assay was performed in the same condition with 4 HAU/well of viruses pretreated with or without Phillyrin or FS21 for 1h at room temperature. Then viral supernatant and cRBC were mixed together at an equal volume of 25μl for another 1h at room temperature. The results were read in the same way as described above.

*Flow cytometry*

Cells were pretreated with Phillyrin or FS21 for 1h and infected with IAV A/Victoria/361/2011 at MOI 5 at 4°C for 1h (viral binding) and incubated at 37°C for additional 1.5h (viral entry). Virus was then removed from cell surface by trypsin. Cells were fixed with 3% paraformaldehyde for 5min and permeabilized with 90% methanol for 10min on ice. Viral particles were stained with FITC conjugated anti-NP antibody (MA1-7322, Theremo Fisher) for 60min. Data were acquired on Canto II cytometer (Becton Dickinson, NJ) and analyzed by FlowJo software (Tree-Star Inc, OR).

*Endosomal acidification*

Endosomal acidification staining was processed as previously described^2^. MDCK cells infected with IAV A/Vic/361/2011were treated with or without Phillyrin or FS21, or bafilomycin A1 (Sigma-Aldrich, positive control) for 1h. Cells were then stained with 4μg/ml acridine orange (Sigma-Aldrich). Fluorescence was detected after excitation at 488nm and merged in two emission windows: 493-560 nm (green) and 590-720 nm (red), using Nikon Elipse Ni-U microscope with Intensilight C-HGFIE.

*NA inhibition*

Neuraminidase (NA) activity was tested by NA assay kit (Fluorometric-Blue, Abcam, IL, ab138888). Virus was pretreated with or without Phillyrin, FS21, or oseltamivir (positive control) for 1h. NA standards or above test sample were mixed with NeuroBlue Indicator assay reaction mixture for 1h. Fluorescence intensity was measured by Wallac Victor (Perkin Elmer, MA) at excitation and emission wavelength of 360/450 nm.

# References

1. ﻿Zheng L, Li H, Fu L, et al. Blocking cellular N-glycosylation suppresses human cytomegalovirus entry in human fibroblasts. Microb Pathog. 2020; 138:103776.

2. Vanderlinden E, Vanstreels E, Boons E, et al. Intracytoplasmic Trapping of Influenza Virus by a Lipophilic Derivative of Aglycoristocetin. J Virol. 2012; 86:9416-31.
